# Niche partitioning and coexistence of two spiders of the genus *Peucetia* (Araneae, Oxyopidae) inhabiting *Trichogoniopsis adenantha* plants (Asterales, Asteraceae)

**DOI:** 10.1371/journal.pone.0213887

**Published:** 2019-10-02

**Authors:** German Antonio Villanueva-Bonilla, Suyen Safuan-Naide, Mathias Mistretta Pires, João Vasconcellos-Neto

**Affiliations:** 1 Programa de Pós-graduação em Biologia Animal, Instituto de Biologia, Universidade Estadual de Campinas, Campinas, SP, Brazil; 2 Instituto de Biociências, Universidade Estadual Paulista Júlio de Mesquita Filho, Campus Botucatu, Botucatu, SP, Brazil; 3 Departamento de Biologia Animal, Instituto de Biologia, Universidade Estadual de Campinas, Campinas, SP, Brazil; Universidad de Sevilla, SPAIN

## Abstract

Niche theory suggests that the coexistence of ecologically similar species in the same site requires some form of resource partitioning that reduces or avoids interspecific competition. Here, from July 2013 to December 2015, we investigated spatial niche differentiation at three different scales of two sympatric congeneric spiders, *Peucetia rubrolineata* and *P*. *flava*, along an altitudinal gradient in shaded and open areas in an Atlantic forest in Serra do Japi, SP, Brazil. These spiders are peculiar in that they present an exclusive association with the plant *Trichogoniopsis adenantha* (Asteraceae). In theory, the coexistence of two *Peucetia* species could be explained by: (1) microhabitat segregation with individuals from different species occupying different parts of the same plants; (2) mesohabitat segregation with different species using plant in different environments; (3) macrohabitat segregation, where different species would not co-occur along the altitudinal gradient. With respect to micro-habitat use, in both species, different instars used different plant parts, while the same instars of both species used the same type of substrate. However, the two *Peucetia* species segregated by meso-habitat type, with *P*. *rubrolineata* preferring *T*. *adenantha* plants in shaded areas and *P*. *flava* preferring those in open areas. Our results support the hypothesis of niche partitioning begetting diversity, and highlight the importance of analyzing habitat use at multiple scales to understand mechanisms related to coexistence.

## Introduction

The mechanisms involved in species coexistence are a central theme in ecology, as they are responsible for maintaining high species diversity in ecosystems worldwide [1–5]). Competitive interactions are stronger between morphologically similar and phylogenetically close sympatric species [6]. The first mathematical models of resource competition proposed that when two species compete for the same resource, one species invariably eliminated the other [7–9]. However, considerable species diversity can be observed coexisting and persisting in the same trophic level of a given web of species interactions [10–12]. Coexistence theory proposes that the coexistence of ecologically similar species at the same site requires a form of resource partitioning [13].

Resource use partitioning has been reported for several taxa, including, mammals [6,14], birds [15–16]), reptiles and amphibians [17–18] fishes [19], and invertebrates [20]. These studies showed that several mechanisms, such as differences in phenology or specific habitat selection, led to a decrease in competition, allowing the species to coexist.

One particular group whose coexistence mechanisms have been given considerable attention are spiders [21–24]. Spiders are one of the most diverse group of terrestrial predators [21,25], with up to 4 species and 131 individuals co-occurring per m^2^ in tropical forests [26–30]. Partitioning of resources among spiders is generally explained by: (1) variation in prey types or sizes as a consequence of different hunting strategies or body size[31–34] reducing interspecific competition and allowing coexistence among species; (2) activity time, with different species varying in phenologies or circadian cycles [34–36] resulting in different population densities of the species throughout the year which reduces interspecific competition; (3) use of space, with different species using different microhabitats as the result of cryptic colorations [37], different foraging behaviors or different sites chosen for the placement of webs (e.g., different heights) [38–42], physiological tolerance [11,43] or based on thermal preference in a gradient of environmental characteristics in cave-dwelling spiders [44–45]. (4) Indirect influence of a species-specific fungus parasite, where the intensity of competition for space is reduced when the dominant host species is highly affected by the parasite and allows the colonization of other less dominant species [46]. (5) Predation, when agonistic interactions (e.g. predation by birds or intraguild predation) determine habitat occupation and niche assembly in tree-dwelling spiders [47]. In the literature, there is controversy over temporal segregation being one of the key mechanisms of coexistence in spider communities. On the one hand, [48] and [49] suggested that temporal segregation is an important factor that reduces niche overlap. [50] also argued that temporal segregation may facilitate the coexistence of species because the peak of abundance of each population will occur at different times, which reduces interspecific competition. For example, the spiders *Meta menardi* and *Metellina merianae* (Tetragnathidae), in addition to presenting distinct abundances throughout the year, also present distinct hunting strategies; *M*. *merianae* combines foraging outside and inside the web whereas *M*. *menardi* feeds exclusively on prey that fall into the web [34]. Temporal segregation may also occur when species differ in periods of activity during the day [51].

Despite the variety of mechanisms mediating coexistence in spiders, only a few studies have assessed different niche dimensions simultaneously in sympatric and ecologically similar species (e.g. [50,52–53]). Understanding how different partitioning mechanisms acting through multiple niche dimensions contribute to the coexistence of ecologically similar organisms can help us advance in the understanding of the processes shaping diversity patterns in local scales. Here we study different aspects of habitat use of two co-occurring spiders in the genus *Peucetia* (Oxyopidae): *Peucetia rubrolineata* Keyserling, 1877 and *P*. *flava* Keyserling 1877. *Peucetia* is a cosmopolitan group, comprising 47 known species, with most species occurring in tropical regions [54–55]. Spiders of this genus do not build webs, weaving silk threads leading to the branches, flowers, or leaves of plants where they live. Some *Peucetia* species were observed showing strong associations with more than 50 species of plants belonging to 17 families, all with glandular trichomes, showing their strong associations with this plant characteristic [56]. Both species *P*. *rubrolineata* and *P*. *flava* occur from Venezuela to Argentina [55,57] and in the Serra do Japi—Brazil, *P*. *rubrolineata* and *P*. *flava* are found exclusively associated with the same plant: *Trichogoniopsis adenantha* (DC) (Asteraceae) [56,58]. The two spiders seem to use the same micro-habitat and food resources [57] and have similar phenologies and population fluctuation through the year [58]. Considering all ecological similarities, this pair of species living in the same plant comprises a promising study system to understand niche partitioning. In this sense the question we address here is: Is there niche partitioning at any level that helps explaining the coexistence of these two species? There are some possibilities that could explain the coexistence of these two *Peucetia* species: (1) microhabitat segregation—whereby *P*. *rubrolineata* and *P*. *flava* would occupy different parts of the plant (e.g., leaves, stems, and flower heads) avoiding competition and allowing coexistence even while inhabiting the same plant. 2) mesohabitat segregation—in which *Peucetia* species exhibit differential distribution based on luminosity and humidity where the host plant is found. 3) macrohabitat segregation—*P*. *rubrolineata* and *P*. *flava* may be distributed differently along an altitudinal gradient. On the one hand, in the field these two species apparently occur in the same specific parts in the host plant (microhabitat), occur in similar proportions along the altitudinal gradient (macrohabitat) and their dynamics and phenologies are similar [58]. On the other hand, these two species of spiders are observed in places with different light levels even if they occupy the same plant, *P rubrolineata* occurring more in shaded places and *P*. *flava* occurring more in open places. Our hypothesis is that there is segregation at a different spatial level (mesohabitat), where one species occurs more often in shaded places and the other species occurs more often in open places due to its physiological tolerances. However, the other possibilities of habitat segregation (micro and macrohabitat) will also be tested in the present study.

## Materials and methods

### Study area

This study was carried out at Serra do Japi, located between 23° 11ʹ S and 46° 52ʹ W of the Atlantic Plateau between the municipalities of Jundiaí, Itupeva, Cabreúva, Pirapora do Bom Jesus, and Cajamar in the state of São Paulo, Brazil. Located between 700 and 1,300m above the sea level, this area (354 km^2^) comprises seasonal mesophyllous forests. The climate is seasonally well defined, with average monthly temperatures varying from 13.5°C in July to 20.3°C in January and with a rainy season in summer (December–March) and dry season in winter (June–August) [59]. Surveys were conducted in different regions of the Serra: (1) at the Department of Water and Sewage (DAE) dam; (2) near the field base at the area locally known as “Biquinha”; (3) along a regional pathway at a locality named “TV cultura” at two altitudes (900–1000 m and 1170–1290 m). To determine the role of (1) microhabitat—stratification on plants, and (2) mesohabitat—patches with different environmental conditions in resource partitioning, studies were conducted from 2013–2015. To determine the role of macrohabitat—altitudinal distribution, research was carried out in March 2014 and March 2017. At the end of the Materials and Methods, there is a table summarizing all methods used, dates, and specific record locations (Table 1).

**Table 1:** Summary of the methodology used to show what was recorded, date and place of registration in Serra do Japi, São Paulo, Brazil.

Segregation type	Definition of segregation type	Methods	Record	Date	Location
Microhabitat	Stratification on plants	• Occurrence of *Peucetia* species in *T*. *adenantha*.	• 300 registered plants in total.	2013–2015	Location 1: DAE (800-900m.a.s.l.). Location 2a: near the research Base (900–1,100m.a.s.l.). Location 2b: pathway "TV culture" (900–1,100m.a.s.l.). Location 3: regional "Tv culture" (1,170–1,290m.a.s.l.).
		• Phenology and availability of types of branches of the plant *Trichogoniopsis adenantha*	• Of the 300 plants previously recorded we selected 20 plants randomly.	2013–2015	Same locations as previously mentioned
		• Distribution of *Peucetia rubrolineata* and *Peucetia flava* instars on *Trichogoniopsis adenantha* branches	• We use the same 300 previously registered plants.	2013–2015	Same locations as previously mentioned
		• Micro-site on the branch of the *Trichogoniopsis adenantha* plant	• We use the same 300 previously registered plants.	2013–2015	Same locations as previously mentioned
		• Similarity in the distribution of instars of the two *Peucetia* species on *Trichogoniopsis adenantha*	• We use the same 300 previously registered plants.	2013–2015	Same locations as previously mentioned
Mesohabitat	Patches with different luminosity	• Plant habitat	• Separate survey, we recorded *T*. *adenantha* plants that had at least one individual of some of the two species of *Peucetia* until reaching a total of 100 records of individuals of each species.	2013–2015	Same locations as previously mentioned
		• Co-occurrence of both spider species	• We use the same 300 previously registered plants.	2013–2015	Same locations as previously mentioned
Macrohabitat	Altitudinal distribution	• Altitudinal distribution	• Record of different number of plants in each location: location 1: 199 plants. Location 2a: 189 plants. Location 2b: 113 plants. Location 3: 115 plants.	March 2014 and March 2017	Same locations as previously mentioned

### Occurrence of *Peucetia rubrolineata* and *P*. *flava* in *Trichogoniopsis adenantha*

**Fig 1:**
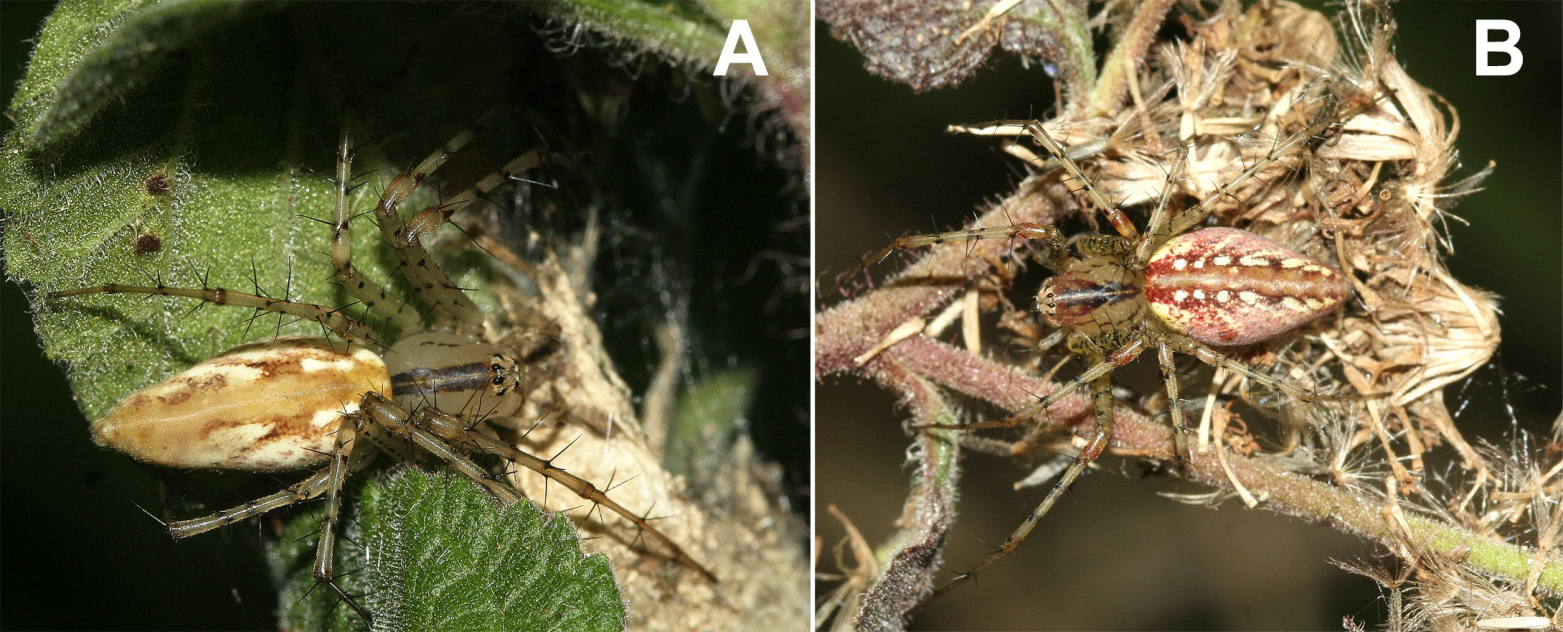
Habits of *Peucetia rubrolineata* and *P*. *flava* (Oxyopidae) registered in *Trichogoniopsis adenantha* (Asteraceae). A) Adult female of *P*. *rubrolineata* with an eggs-sac inside the shelter. B) Adult female of *P*. *flava* on a dry branch.

During the period between July 2013 to December 2015, we inspected 300 *T*. *adenantha* in open and shaded areas (S1 Table). For each plant with spiders, we registered the species of spider, the instar of development and the specific part in the plant where the spider was found. In each monthly survey the 300 plants were randomly selected so that these 300 plants could be different each month. The two species of *Peucetia* can be easily recognized in the field by the different pattern of staining on the cephalothorax and abdomen. In *P*. *rubrolineata* a continuous double line can be observed in the cephalothorax and in *P*. *flava* that same line is cracked (Fig 1). We identified the instars in accordance with the guidelines published by [60]. This classification was based on the size of the cephalothorax and the first pair of legs (For details see [58,61]. We collected some individuals from both species of *Peucetia* for later corroboration of identification with the specialist (Brescovit A.D.),

### Microhabitat—Stratification on plant

#### Phenology and availability of types of branches of the plant *Trichogoniopsis adenantha*

To uncover the phenology of *T*. *adenantha*, of the 300 plants previously recorded we selected 20 plants randomly in each month and registered the number of vegetative branches and the state of development of flower heads. Once the phenology of the plant was recorded, we could evaluate if the phenology affected the occurrence of instars of *P*. *rubrolineata* and *P*. *flava* on different branches of the host plant.

We classified flower heads in situ into five phenophases as described by [62] and [63]: (F1) closed bud, very small, bracts covering the whole bud; (F2) open bud, with all flowers visible but all closed (pre-anthesis); (F3) open flowers with long and bluish-pink stigmas (anthesis and fertilization); (F4) complete formation of yellow flowers on flower head; stigmas beginning to drop (fruit development phase); and (F5) dry flower head; mature seeds, beginning of the dispersion phase (S2 Table).

Subsequently, based on the predominance of the phenophases of each flower head, we classified the branches into the following categories: (1) vegetative branches; (2) branches with flower buds, with the mean number of flowers in the F1 or F2 phase greater than 50% of the total number of flower heads (owing to inter-individual variation in the number of flower heads from 6 to 18 flower heads per branch); (3) branches with open flowers, with the mean number of flowers in the F3 greater than 50% of the total number of flower heads; (4) type 4 branches, with the mean number of flowers in the F4 greater than 50% of the total number of flower heads; (5) type 5 branches, with the mean number of flowers in the F5 greater than 50% of the total number of flower heads.

#### Distribution of *Peucetia rubrolineata* and *Peucetia flava* instars on Trichogoniopsis adenantha branches

To verify whether the distribution of the *P*. *rubrolineata* instars was similar in the different types of branches, we compared the frequency of the types of branches available with the branches actually occupied by the instars of the spiders. For this analysis, we used only the information from the months where *P*. *rubrolineata* was most abundant within the two-and-a-half-year period previously recorded. The same procedure was performed for *P*. *flava*.

#### Micro-site on the branch of the *Trichogoniopsis adenantha* plant

To verify whether instars of *P*. *rubrolineata* have the same pattern of distribution in different specific parts of the plant (leaf, stem, and flower head and dry flower head), we used the Chi-square Test. Here again, we used the data from the same two-and-a-half-year period previously recorded. As the null hypothesis, we predicted the spiders of each instar would occur on similar parts of the branch with similar probability. We also adopted the same protocol for *P*. *flava*.

#### Similarity in the distribution of instars of the two *Peucetia* species on *Trichogoniopsis adenantha*

We also investigated whether the same instars of both species occupy the same part of the plant and used the Chi-squared Test to examine the significance of observed differences in the frequency of occupation. To do this, we related the total available parts of the inspected plants to the parts that were actually occupied by the same instar of both *Peucetia* species.

### Mesohabitat—Patches with different environmental conditions

#### Plant habitat

In a separate survey, we recorded *T*. *adenantha* plants that had at least one individual of some of the two species of *Peucetia* until reaching a total of 100 records of individuals of each species. In those plants in which an individual of *Peucetia* was registered we set a camera with a fisheye lens to photograph the cover vegetation above each plant. Subsequently, we divided photographs into sectors and classified each site according to canopy cover percentage classes: 0–20%; 21–40%; 41–60%; 61–80%, and 81–100%. We then tested if the distribution pattern of the two species of *Peucetia* differed among classes of vegetal cover, using a Chi-squared Test.

#### Co-occurrence of both spider species

To determine the degree of niche overlap of the two species of *Peucetia* in each instar, we used the Jaccard overlap index [64]:

$$J=\frac{A}{A+B+C}$$

Where "A" is the number of plants where both species of spiders were present simultaneously, "B" is the number of plants where only *P*. *rubrolineata* was present and "C" the number of plants where only *P*. *flava* was present. To calculate the index, we pooled together all the surveyed plants where at least one individual of some of the spider species was present during the two-and-a-half-year period. The index was calculated for the same instar of the two species of *Peucetia* separately because the two species presented in similar proportions of abundance throughout the year and the same instars of the two species occupy the same type of branch on the plant.

### Macrohabitat—Altitudinal separation

#### Altitudinal distribution

We verified whether the species of *Peucetia* had a differential altitudinal distribution pattern. Here, *T*. *adenantha* plants were inspected in March 2014 at four different sites with different altitudes: (1) first site called dam of the DAE with an altitude of 800–900 m; (2) this second location at an elevation of 900–1,100 m was subdivided into two locations which have the same altitude, (2a) region near the research base that has a shaded area and (2b) pathway of “TV culture” that has an open region; (3) third location regional "TV culture" at an altitude of 1,170–1,290 m. In each altitudinal range, we inspected the *T*. *adenantha* plants in open and shaded areas as follows: (1) in the first location we registered 199 plants (149 and 50 plants in shaded and open areas, respectively); (2a) in this location was registered 189 plants (29 and 160 plants in shaded and open areas, respectively);(2b) in this location was registered 113 (98 and 15 plants in shaded and open areas, respectively); (3) finally, in this location was registered 115 plants (20 and 95 plants in shaded and open areas, respectively). The numbers of plants available for inspection varied at different altitudes. In relation to the site (2a) that presents an altitude range of 900–1,100 m, in the year in which the sampling was re-sampled, in 2014, it was an open area due to the mixed vegetation of native trees and *Eucalyptus* had been cut and, consequently, *T*. *adenantha* plants were more abundant in these open areas. Three years later (2017), the tree vegetation grew and shaded most of the *T*. *adenantha*. Thus, we sampled this area (2a) again in 2017 to verify the effect of shading on the relative abundance of the two *Peucetia* species. In each *T*. *adenantha* observed, we recorded the environment in which the plant was (open or shaded according to the photographs of canopy cover) and the *Peucetia* species found on each plant. We used an altimeter to measure altitude. Subsequently, we applied the Chi-squared test (with Williams' correction) to compare the frequencies of the two spider species with the frequencies of *T*. *adenantha* in open or shaded environments at different altitudes. We conducted these analyses to separate the effect of altitude with respect to the occurrence of these spiders in different microenvironments, in the case of open areas and more shaded areas.

For all comparisons regarding the frequencies of use of habitat—temporal segregation, ontogenetic variation, microhabitat segregation and macrohabitat partitioning—we used a Chi-square test with Monte Carlo permutations with 10000 simulations [65] to test for significance considering an alpha value of 0.05. In cases of multiple pairwise comparisons we used the Williams´ correction. For all statistical analyzes, we used the free software R [66]. The package “rmngb” was used for all Chi-squared analyses.

## Results

### Phenology of *Trichogoniopsis adenantha*

**Fig 2:**
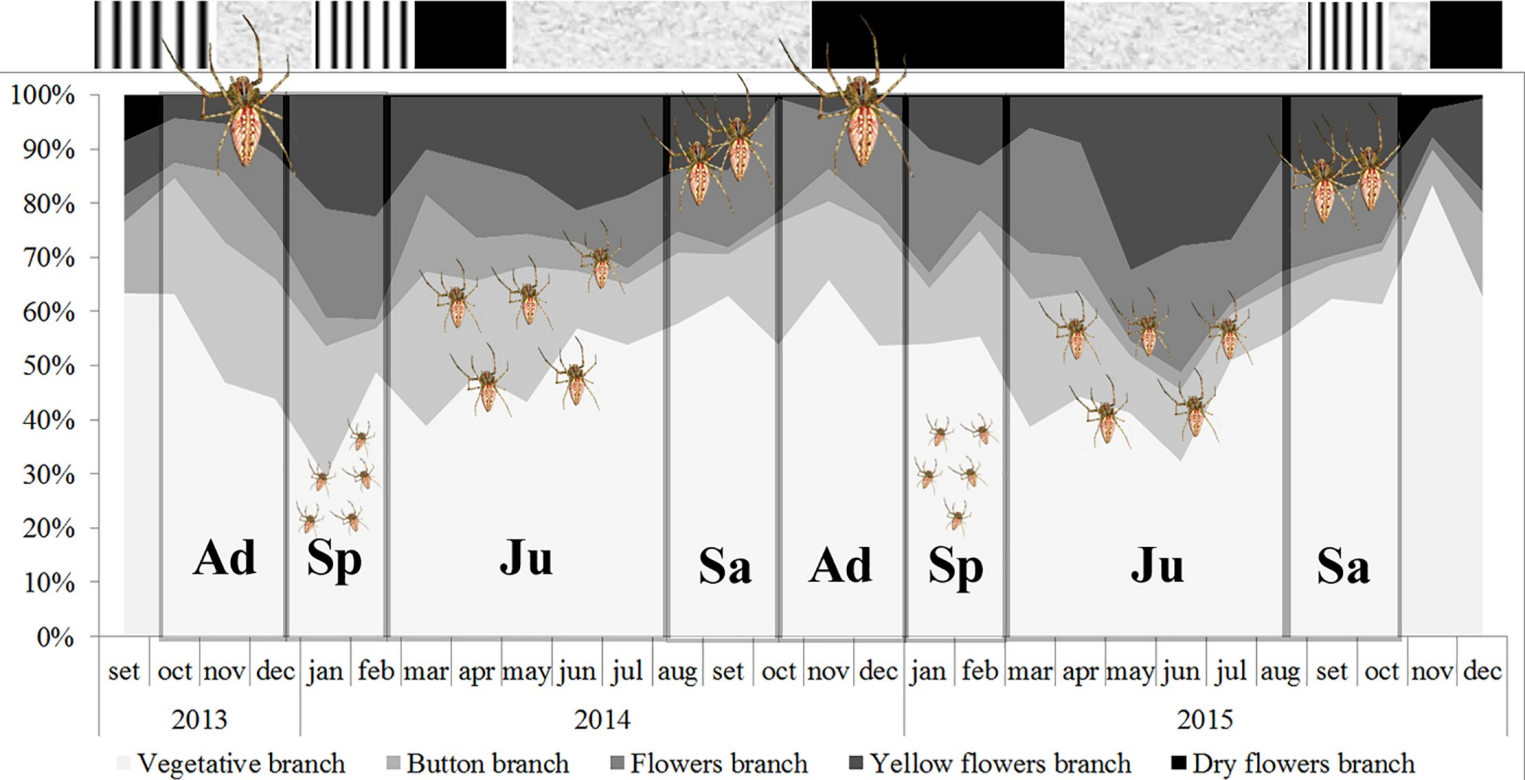
Phenogram of the different types of *T*. *adenantha* branches with the representation of the different age classes of *Peucetia* (spider silhouettes in different sizes) in the type of branch where they are frequently found. **Ad** = adult individuals; **Sp** = Spiderlings; **Ju** = Young and Juvenile; **Sa** = subadult individuals. The upper bar of the graph represents the rainy periods where: Grey spaces indicate dry period; spaces with black vertical bars indicate period with average rainfall; Black spaces indicate rainy season. Young and juveniles have been put together in the diagram because the use of parts of the host plant are basically the same.

In general, *T*. *adenantha* had both vegetative and reproductive branches throughout the year; however, the proportions varied with the season (Fig 2). In winter and spring, vegetative branches occurred in larger proportions. At the end of the dry season, usually in spring (months), there was growth of vegetative branches, followed by an increase in the number of reproductive branches, with a large number of buds in spring and summer. During March and April, there was a period with greater abundance of branches with open flowers. In May, a large number of flowering branches occurred in phase 4 (seed formation), and in June and July, there were several flowering branches in phases 4 and 5 dry (flower heads). In general, vegetative branches were more abundant than reproductive branches. Production of flower heads occurred throughout the year; however, the proportion of each phenophase varied from year to year (Fig 2).

### Microhabitat—Stratification on the plant

#### Distribution of the *Peucetia* species on *T*. *adenantha* branches types

**Fig 3:**
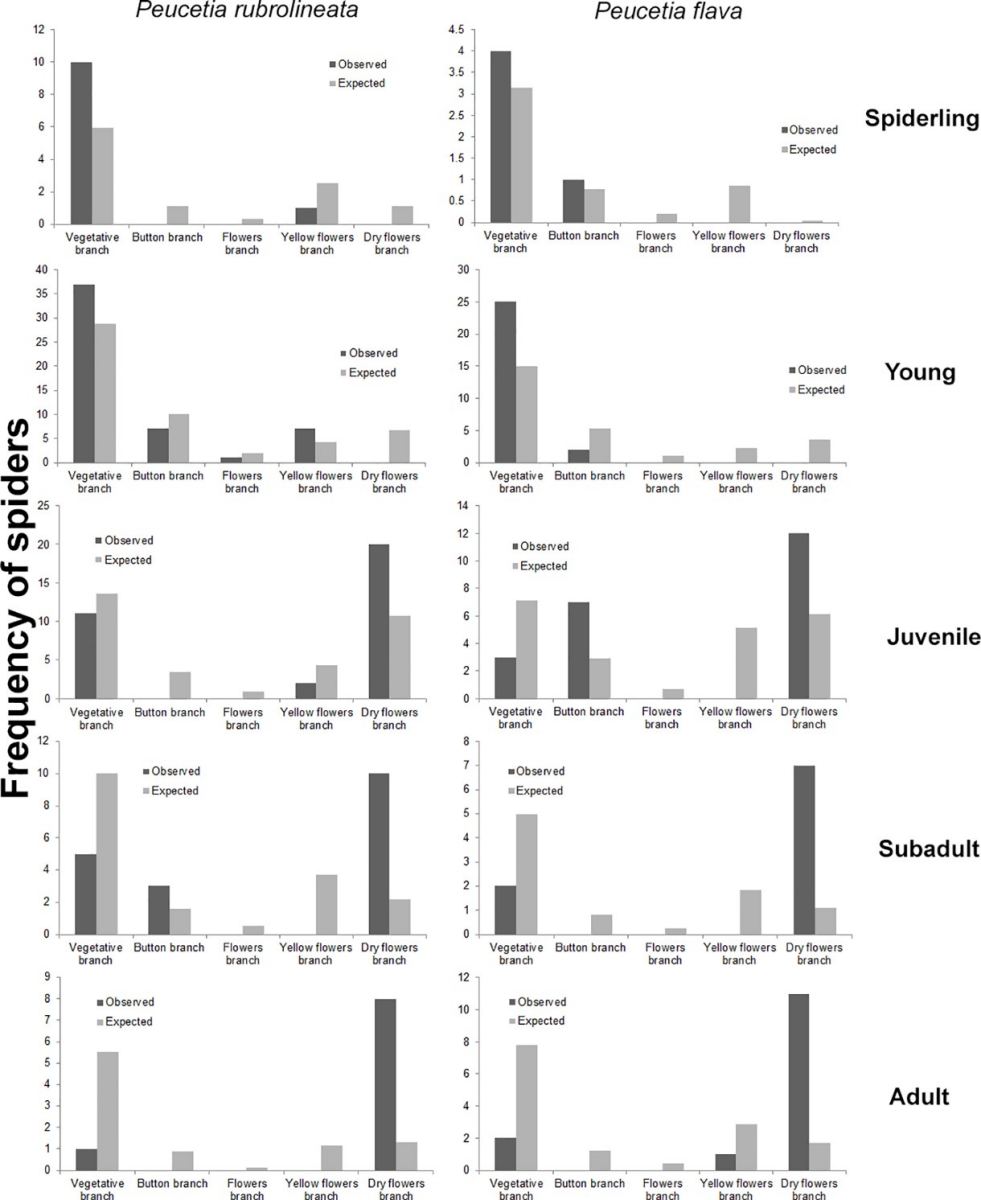
Observed and expected frequencies of the *P*. *rubrolineata* e *P*. *flava* instars on different types branches of *T*. *adenantha*.

Young spiders of both species were more frequent on the vegetative branches than in reproductive ones (Chi-squared test = *P*. *rubrolineata*: χ^2^ = 9.37, *P* = 0.034; *P*. *flava*: χ^2^ = 10.777, *P* = 0.005; in 10000 simulations). Juvenile spiders were more frequent on vegetative branches and on type five branch (Chi-squared test = *P*. *rubrolineata*: X^2^ = 8.31, *P* = 0.043; *P*. *flava*: X^2^ = 11.111, *P* = 0.009), as well as subadults, (Chi-squared test = *P*. *rubrolineata*: X^2^ = 11.327, p = 0.005; *P*. *flava*: X^2^ = 8.512, p = 0.006) although the other types of branches are more frequent. Adult individuals were more frequent on type 5 branches, especially in the inflorescences where shelters made with leaves and dry flower heads are often observed (Chi-squared test = *P*. *rubrolineata*: X^2^ = 10.096, *P* = 0.0038; *P*. *flava*: X^2^ = 12.791, *P* = < 0.001). Finally, spiderlings of the two *Peucetia* species were the only ones that presented similar observed and expected frequencies in all types of branches (Chi-squared test = *P*. *rubrolineata*: X^2^ = 4.22, *P* = 0.215; *P*. *flava*: X^2^ = 1.22, *P* = 1) (Fig 3). Regardless of the proportion of the different branches, the pattern of distribution of the instars of the two species of *Peucetia* in these branches remained the same throughout the year.

#### Micro-sites on the branches of *Trichogoniopsis adenantha* plants

**Fig 4:**
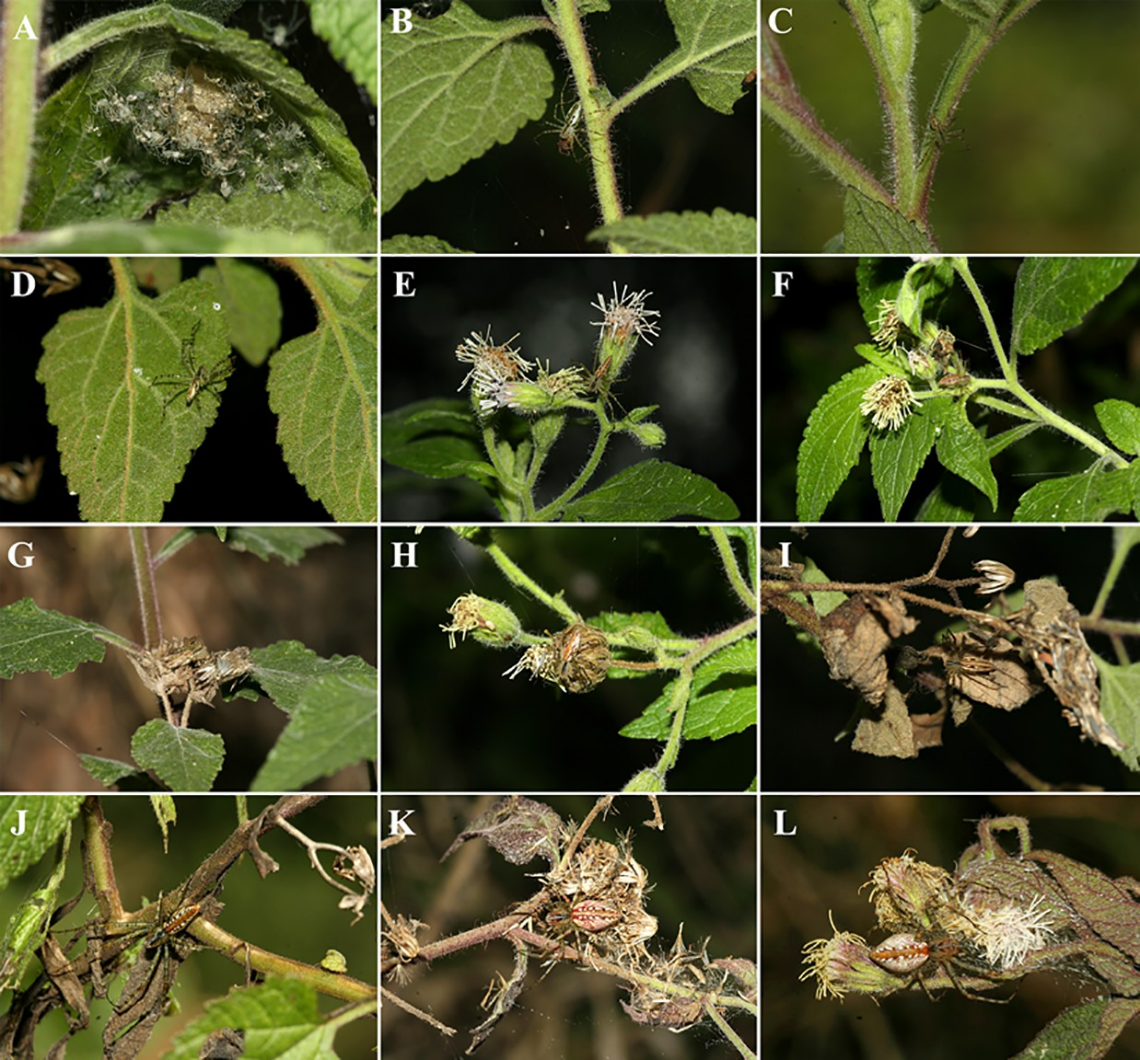
Instars of *Peucetia rubrolineata* and *P*. *flava* on different parts of the plant *Trichogoniopsis adenantha* (Asteraceae) where they are frequently observed (leaf, stem, flower heads, flower, shelter of dry flower heads). A = second instar *P*. *rubrolineata*; B-D = third instar *P*. *rubrolineata*; E = fourth instar *P*. *rubrolineata*; F = fourth instar *P*. *flava*; G-H = fifth instar *P*. *rubrolineata*; I = sixth instar *P*. *rubrolineata*; J = seventh instar (subadult) *P*. *flava*; K-L = eighth instar (adult) *P*. *flava*.

The distribution of different instars of both *P*. *rubrolineata* and *P*. *flava* on different parts of the branches was inhomogeneous (Table 2). Second and third instars of *P*. *rubrolineata* were more frequently recorded on leaves, whereas fourth-instar spiders were observed on the leaves, stem, and flower heads, the fifth, sixth, and seventh instars occurred more frequently in the dry flower heads. Adults (eighth-instar) occurred more frequently in dry flower heads and in shelters made from remnants of the dry flower heads (Table 2; Fig 4).

**Table 2:** Abundance of spiders of *Peucetia rubrolineata* and *P*. *flava* (Oxyopidae) in different parts of the plant *Trichogoniopsis adenantha* (Asteraceae).

Peucetia rubrolineata								
Instar	Leaf	Stem	flower heads	Dry flower heads	Shelter	Total	X^2^-test	P value
2°	21	5	1	2	0	29	19.72	< **0.001**
3°	84	31	22	14	0	151	62.78	< **0.001**
4°	49	22	25	30	0	126	33.46	< **0.001**
5°	47	9	23	111	0	190	96.3	< **0.001**
6°	25	5	12	79	1	122	67.42	< **0.001**
7° (subadult)	10	13	4	34	1	62	24.32	< **0.001**
8°(adult)	8	1	0	10	37	56	34.13	< **0.001**
*Peucetia flava*								
Instar	Leaf	Stem	flower heads	Dry flower heads	Shelter	Total	X^2^-test	P value
2°	10	8	6	0	0	24	12.36	**0.0061**
3°	49	21	6	9	0	85	40.65	< **0.001**
4°	42	28	20	15	0	105	30.02	< **0.001**
5°	32	8	18	91	0	149	76.36	< **0.001**
6°	18	6	14	77	2	117	60.01	< **0.001**
7° (subadult)	11	1	3	31	1	47	28.54	< **0.001**
8°(adult)	15	1	3	20	50	89	41.20	< **0.001**

Bold values indicate significant values.

**Fig 5:**
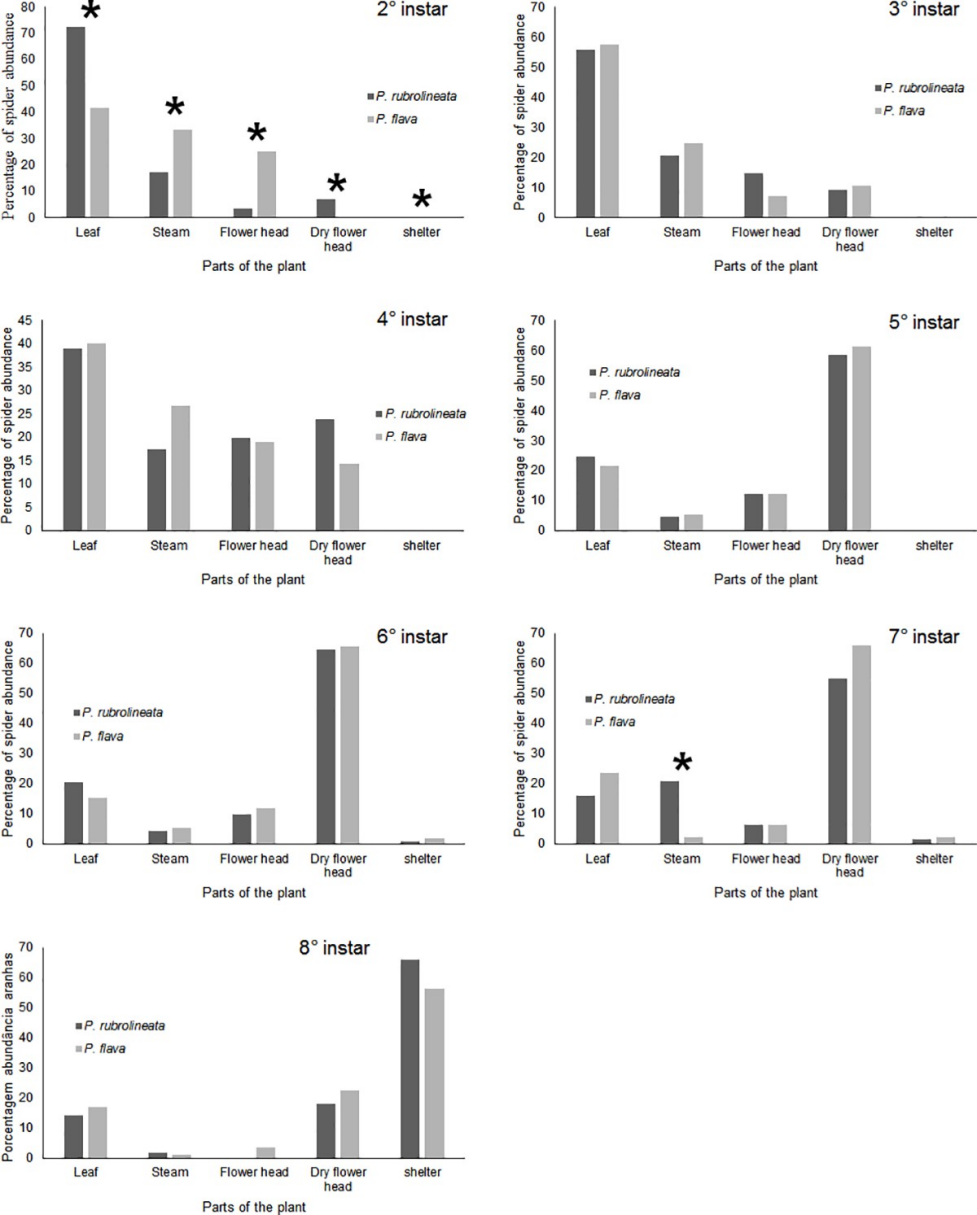
Comparison of the observed frequency of developmental instars of *Peucetia rubrolineata* and *P*. *flava* (Oxyopidae) on the different parts of the plant *Trichogoniopsis adenantha* (Asteraceae) in Serra do Japi, SP. Brazil. Instar 7° = subadult; Instar 8° = adult. Asterisks indicate statistically different values.

Despite the difference in the occupation of the types of branches and parts of the plant among instars of the same species, when the same instars of the two species are compared, the pattern of occupation on the plant is similar (Table 3; Fig 5). Only spiderlings presented a different occupation pattern between species; *P*. *rubrolineata* spiderlings used preferentially the leaves, whereas *P*. *flava* spiderlings were mostly found in the stem and flower heads (Table 3; Fig 5).

**Table 3:** Comparison of the percentage of occupation of the parts of the plant by each of the instars of the two species of *Peucetia*. Bold values indicate significant values.

**Part of the plant**	**2°**			
	***P*. *rubrolineata***	***P*. *flava***	**X**^**2**^**-test**	**p value**
Leaf	72.41	41.66	8.2	**0.004**
Stem	17.24	33.33	5.1	**0.02**
Flower head	3.44	25.00	16.3	< **0.001**
Dry flower head	6.89	0	3.4	**0.06**
Shelter	0	0	0	1
	**3°**			
	***P*. *rubrolineata***	***P*. *flava***	**X**^**2**^**-test**	**p value**
Leaf	55.62	57.64	0.03	0.85
Stem	20.52	24.70	0.38	0.53
Flower head	14.56	7.05	0.26	0.10
Dry flower head	9.27	10.58	0.08	0.76
Shelter	0	0	0	1
	**4°**			
	***P*. *rubrolineata***	***P*. *flava***	**X**^**2**^**-test**	**p value**
Leaf	38.88	40	0.01	0.9
Stem	17.46	26.66	1.92	0.16
Flower head	19.84	19.04	0.01	0.89
Dry floer head	23.80	14.28	0.23	0.12
Shelter	0	0	0	1
	**5°**			
	***P*. *rubrolineata***	***P*. *flava***	**X**^**2**^**-test**	**p value**
Leaf	24.73	21.47	0.23	0.63
Stem	4.73	5.36	0.04	0.84
Flower head	12.10	12.08	< 0.001	0.99
Dry flower head	58.42	61.07	0.05	0.80
Shelter	0	0	0	1
	**6°**			
	***P*. *rubrolineata***	***P*. *flava***	**X**^**2**^**-test**	**p value**
Leaf	20.49	15.38	0.72	0.39
Steam	4.09	5.128	0.11	0.73
Flower head	9.83	11.96	0.20	0.64
Dry floer head	64.75	65.81	0.009	0.92
Shelter	0.81	1.70	0.31	0.57
	**7°**			
	***P*. *rubrolineata***	***P*. *flava***	**X**^**2**^**-test**	**p value**
Leaf	16.12	23.40	1.33	0.24
Steam	20.96	2.12	15.36	**< 0.001**
Flower head	6.45	6.38	< 0.001	0.98
Dry floer head	54.83	65.95	1.02	0.31
Shelter	1.61	2.12	0.07	0.79
	**8°**			
	***P*. *rubrolineata***	***P*. *flava***	**X**^**2**^**-test**	**p value**
Leaf	14.28	16.85	0.21	0.64
Steam	1.78	1.12	0.15	0.69
Flower head	0	3.37	1.68	0.19
Dry floer head	17.85	22.47	0.52	0.46
Shelter	66.07	56.17	0.8	0.37

### Mesohabitat—Patches with different environmental conditions

#### Co-occurrence of both *Peucetia* species

**Fig 6:**
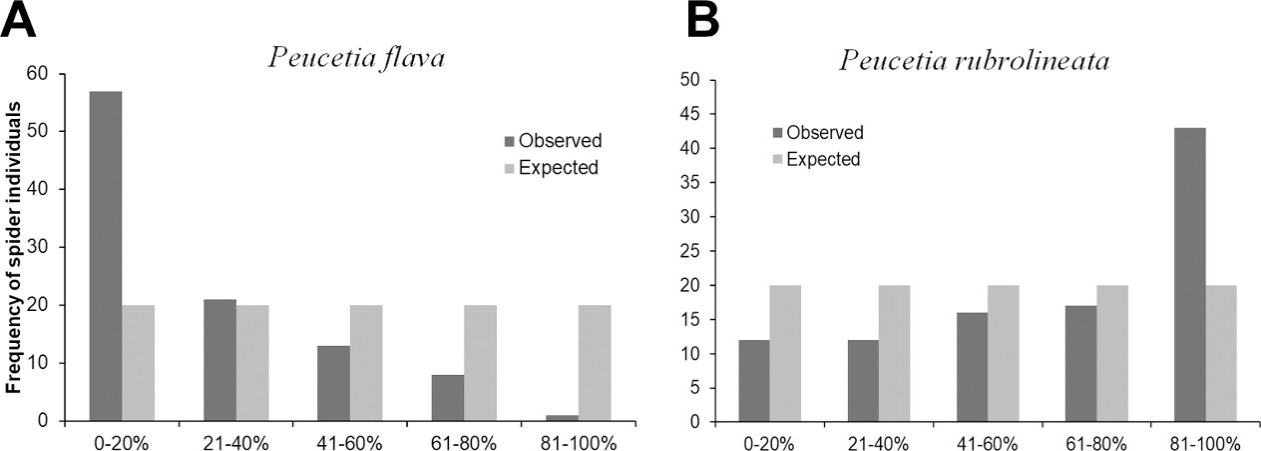
Distribution of *Peucetia rubrolineata* and *P*. *flava* on *Trichogoniopsis adenantha* plants in environments with different vegetation cover (%). Observed and expected frequencies of A) *P*. *flava* and B) *P*. *rubrolineata* in different percentages of vegetation cover.

The frequencies of the two species of *Peucetia* were different in the two types of vegetation cover (X^2^ = 75.444, *P* = < 0.001; in 10000 simulations) (Fig 6). *Peucetia rubrolineata* was recorded at a higher frequency in *T*. *adenantha* plants located in environments with denser canopy (X^2^ = 41.622, *P* = < 0.001) (Fig 6B). Conversely, *P*. *flava* was recorded more frequently in *T*. *adenantha* plants in more open environments (X^2^ = 13.085; *P* = 0.01) (Fig 6A). In the intermediary levels of vegetation cover, the frequencies of both *Peucetia* species were similar (Fig 6).

In environments with intermediate canopy cover, a larger number of plants were observed with the two *Peucetia* species. Of the 729 plants that had spiders during the study period, 43.5% contained only *P*. *flava* and 53.3% contained only *P*. *rubrolineata*, whereas only 3.4% of the plants contained both species. The values of niche overlap varied from 0.024 to 0.059, with a mean of 0.034, indicating low overlap between the same instar of the two species of *Peucetia* (Table 4).

**Table 4:** Niche overlap index between the same instar of the two species of *Peucetia* and the average value of niche overlap.

Instars	Total plants with spiders	Plants with two spider species	Niche overlap index
2°	36	0	0
3°	163	4	0.024
4°	108	4	0.037
5°	135	8	0.059
6°	113	3	0.026
7°	77	2	0.025
8°	97	4	0.041
		Average value	0.034

### Macrohabitat—Altitudinal separation

#### Altitudinal distribution

Both *Peucetia* species were found at all altitudes at the study site, but in different frequencies (63 individuals of *P*. *rubrolineata* and 37 individuals of *P*. *flava* in total). However, these frequencies varied depending on availability of *T*. *adenantha* plants in more open areas (low canopy cover) or in more closed places (high canopy cover) and not with respect to the altitude. Sites with low canopy cover had mostly individuals of *P*. *flava*, whereas sites with high canopy cover had individuals of *P*. *rubrolineata* (Table 5). In the observation made in 2014 at site (2b), called the TV Cultura Pathway Region, *T*. *adenantha* plants were found with little canopy cover and a higher abundance of *P*. *flava* was recorded. Three years later, in 2017, a higher abundance of *P*. *rubrolineata* was observed (Table 5) in the same region and at the same altitude (2b*), when vegetation grew and canopy cover increased on *T*. *adenantha* plants.

**Table 5:** Comparison of the frequencies of *P*. *rubrolineata* and *P*. *flava* on *Trichogoniopsis adenantha* in shaded and open areas.

Altitude m	Plants in Shaded areas	Plants in open areas	Total of plants	Plants with *P*. *rubrolineata*	Plants with *P*. *flava*	X^2^ Test	P value
(1) 800–900	149	50	199	20	5	0.179	0.74
(2a) 900–1100	98	15	113	13	4	0.594	0.64
(2b) 900–1100	29	160	189	1	13	0.473	0.59
(2b*) 900–1100	95	30	125	27	9	0.009	1
(3) 1170–1290	20	95	115	2	6	0.13	1

DAE dam, (2a) Region of Base, more shaded, record made in 2014. (2b) Region path of TV culture, more open, record made in 2014. (2b*) Same Region path of TV culture, record made in 2017 (more shaded), (3) Region path of TV culture at highest altitude, record 2014.

## Discussion

Niche theory suggests that the coexistence of ecologically similar species requires some form of resource partitioning that reduces or prevents interspecific competition [13]. In the studied system, the two *Peucetia* species inhabiting plants of *T*. *adenantha* present similar phenology and population dynamics [58]. In addition, the same instars of the two spider species had similar distributions across the different parts of the plant, indicating that the microhabitat use overlaps between the two species. However, our hypothesis that the two *Peucetia* species inhabiting *T*. *adenantha* plants are segregated has been confirmed, because they were distributed differently at the mesohabitat level. *Peucetia rubrolineata* occurred more frequently in places where canopy cover was high, whereas *P*. *flava* occurred more frequently in sites with open canopy. Although several studies have shown that interspecific competition is one of the main factors frequently observed in phylogenetically close spiders (e.g. [41]), in our study there is low overlap at the mesohabitat scale. Thus, we can infer from our results that competition may not be an important factor currently shaping these populations, although competition in the past might have favored habitat segregation patterns. Further research on sites where these species do not occur in sympatry could shed light on whether this segregation is a product of interspecific interactions or simply reflect different responses to abiotic conditions.

Results also indicated that these species have different altitudinal distributions because *P*. *rubrolineata* was more abundant at lower altitudes in the Serra do Japi, whereas *P*. *flava* occurred more frequently at higher altitudes. However, this difference seems to be more related to the availability of plants in different environments (shaded and exposed) than related to the altitude. At higher altitudes, the frequency of plants in the sunny areas was higher, as was the frequency of *P*. *flava*. In contrast, at lower altitudes, shaded areas were more frequent and the frequency of *P*. *rubrolineata* was higher. At intermediate altitudes, the abundance of spiders depended on the abundance of plants in the sun and shade. At the same site, the abundance of *P*. *flava* and *P*. *rubrolineata* varied in different years depending on the degree of shading. Although the two *Peucetia* species occurred in environments with intermediate canopy cover, the co-occurrence of these spiders in the same host plant was infrequent. Although we observed a clear distribution pattern of the two species of *Peucetia* more related to the type of environment (open or shaded) than to altitude, this statement should be taken with caution, because the recorded abundances of the two species for each altitude were low. Future studies would be necessary where the number of spiders collected by altitude was higher, and the low influence of altitude on the coexistence of the two species of *Peucetia* was corroborated.

Although *P*. *rubrolineata* and *P*. *flava* are sympatric and their population dynamics are similar throughout the year, the general pattern of spatial segregation registered occurred in the two years of study. This type of segregation as a central mechanism in the coexistence of sympatric spiders has been recorded in other cursorial spiders of the genus *Syspira* (Miturgidae), where two species of Miturgidae also exhibited different spatial distribution related to differences in temperature and humidity at the site [67]. In a central Amazon forest, [50] recorded significant differences in the soil type preference (sandy and clay soils) of four species of *Ctenus* (Ctenidae). In Lycosidae, horizontal stratification appears to be a common mechanism of niche differentiation [68]. In the temperate region the relative abundance of *Pardosa alacris* gradually increased with the canopy opening (mesohabitat level), whereas the opposite trend was observed for *P*. *lugubris*. Thus, niche differentiation along the canopy opening gradient seems to mediate the coexistence of these two species in meta-communities [52]. The Lycosids *Geolycosa xera archboldi* and *G*. *hubbelli* also exhibited preferences for different types of vegetation (mesohabitat level) and for different percentage of litter cover (micro habitat level) [69]. These examples show that habitat segregation at the mesohabitat level, as we found here, may be an important mechanism maintaining the high diversity of arthropods, especially spiders, in forested habitats.

Spiderlings of the two *Peucetia* species were more frequent on the vegetative branches of *T*. *adenantha*. A number of predatory arthropods (e.g., others *Peucetia* spider species, the spider *Misumenops argenteus*, Thomisidae) are specifically associated with glandular trichome-bearing plants like *T*. *adenantha*, where they capture prey attached to the sticky thricomes [57,70]. Spiderlings are probably more frequent in plant parts with more trichomes, which would increase the number of prey trapped [71]. Moreover, trichomes provide some protection to the spiderlings from their enemies, such as other spiders [72]. Fourth-instar young spiders were observed preying on endophytic herbivores of the flower head of *T*. *adenantha*, especially the fruit flies *Trupanea* sp. (Tephritidae), whereas juveniles (fifth and sixth instars) consumed inchworms of Geometridae that feed on flower heads and other insects that inhabit the plant (see [60]). In the case of subadults and adult spiders, they occurred more frequently in the flower head of the plant, especially those branches that also have dry structures where they rest. This higher frequency likely occurred because, besides having a place for camouflaging, it is also easier for them to capture larger prey, such as floral visitors, which visit flower heads with open flowers at phase 3 and phase 4. In fact, [71] recorded adult spiders feeding on *Pseudoscada erruca*, *Aeria olena*, and *Episcada carcinia* (Ithomiinae), and all floral visitors on the flowers of *T*. *adenantha*. Additionally, adult females build the nest to place the egg sac using the plant's dried structures (personal observation). Thus, preferences for types of branches could be partially explained by the types of prey available and used as food by different instars as well as reproductive behavior. This ontogenetic habitat segregation found in both of the studied species may also decrease intraspecific competition among different instars of the same species, which would be reflected in the success of individuals in different stages of development.

Finally, because these two species of *Peucetia* are associated exclusively with *T*. *adenantha*, we expect that in the other parts of the Serra where we did not register these species, the same pattern of distribution and the same degree of spatial overlap occur in the different scales that we study. However, these species of *Peucetia* may be associated with other plants that have glandular trichomes, so if there are other plants in other places with glandular trichomes there would probably be other distribution patterns. However, further studies would be needed to compare the degree of effect of the availability of other plants on the distribution of *Peucetia*. Examining the niche partition between two morphologically similar, sympatric spiders, with overlapping temporal distributions we show inter- and intraspecific segregation in habitat use at different spatial scales. Although *P*. *flava* and *P*. *rubrolineata* are occurring in sympatry with similar phenologies, at similar altitudes and using the same parts of the same plants species, the distribution of the *T*. *adenantha* plant in shaded and open environments affects the distribution of the two species corroborating our hypothesis. Our results support the hypothesis of niche partitioning begetting diversity, and highlight the importance of analysing habitat use at multiple scales to understand mechanisms related to coexistence.

## Supporting information

S1 Table Phenology and abundance *Peucetia* species. This data set is about the phenology and abundance of the two species of *Peucetia* over the two and a half years. Each month we register 300 plants of *Trichogoniopsis adenantha* randomly to register the spiders. We recorded for each spider the developmental stage, sex and part of the plant where the individual was.
(TXT)

S2 Table Phenology *Trichogoniopsis adenantha* plants. This set of data is on the phenology of *T*. *adenantha* plant. Of the 300 plants recorded, we randomly selected 20 plants each month to record the penology. In each registered plant the number of vegetative and reproductive branches was noted. Moreover, in each reproductive branch we note the phenophase of the flower heads.
(TXT)
